# Single-cell mass cytometry cartography highlights systemic lupus erythematosus and Sjögren’s disease immune differences

**DOI:** 10.3389/fimmu.2026.1834700

**Published:** 2026-10-01

**Authors:** Franziska Szelinski, Ana-Luisa Stefanski, Annika Wiedemann, Hector Rincon-Arevalo, Eva Schrezenmeier, Tobias Alexander, Van Duc Dang, Andreas Frei, Andreia C. Lino, Thomas Dörner

**Affiliations:** 1 Deutsches Rheuma-Forschungszentrum, a Leibniz Institute, Berlin, Germany; 2 Department of Rheumatology and Clinical Immunology, Charité Universitätsmedizin Berlin, corporate member of Freie Universität Berlin and Humboldt-Universität zu Berlin, Berlin, Germany; 3 Department of Nephrology and Medical Intensive Care, Charité Universitätsmedizin Berlin, Corporate Member of Freie Universität Berlin and Humboldt-Universität zu Berlin, Berlin, Germany; 4 Grupo de Inmunología Celular e Inmunogenética, Facultad de Medicina, Instituto de Investigaciones Médicas, Universidad de Antioquia UdeA, Medellín, Colombia; 5 Roche Innovation Center Basel, Basel, Switzerland

**Keywords:** pSjD, pSS, SLE, B cells, T cells, innate cells, mass cytometry

## Abstract

**Objectives:**

Autoimmune diseases such as primary Sjögren’s disease (pSjD) and systemic lupus erythematosus (SLE) share clinical and serological features, including type I interferon signatures, autoantibody formation, and chronic inflammation, but the underlying immunopathology is not the same. To support the development of targeted therapies, we aimed to systematically characterize disease-specific alterations in peripheral immune cell subsets of pSjD and SLE compared to healthy donors (HD).

**Methods:**

We analyzed peripheral blood mononuclear cells from 15 HD, 12 pSjD and 21 SLE patients by mass cytometry. We manually pre-gated viable CD19^+^ B cells, CD3^+^ T cells, and innate immune populations before performing high-dimensional, consensus clustering. We compared relative subset frequencies and marker expression, including costimulatory and inhibitory receptors, between the three cohorts.

**Results:**

In the B cell compartment, we identified 13 clusters. SLE patients showed an relative expansion of plasmablasts and two double-negative (IgD^−^CD27^−^) B cell clusters, one CD21^−^ and one CD19^low^ with high IgA. Both had an activated phenotype with elevated CD38, CD86, and Ki-67. In SLE, the costimulatory molecules CD86 and CD226 were upregulated on memory and DN B cells. By contrast, B cell changes in pSjD were more modest. We found 12 T cells clusters. Both diseases had increased CD4^+^ PD-1^+^ ICOS^+^ circulating T follicular helper-like cells, and pSjD showed expansion of CD8^+^ effector memory (Tem) and terminally differentiated (Temra) T cell subsets. Relative innate cell frequencies did not differ substantially between groups, however, intermediate CD226^hi^ monocytes were reduced in SLE but showed higher activation marker expression (CD86, HLA-DR). Network correlation analysis revealed higher immune‐cell connectivity in SLE compared to pSjD.

**Conclusion:**

SLE is characterized by B cell activation, plasmablast expansion, and costimulatory upregulation, while pSjD is mainly defined by alterations in the CD8^+^ T cell compartment. These disease‐specific immunologic signatures highlight distinct patterns in SLE and pSjD and generate hypotheses for future studies investigating targeted therapeutic approaches.

## Introduction

Primary Sjögren’s disease (pSjD) and systemic lupus erythematosus (SLE) are both inflammatory rheumatic diseases with unknown and multifactorial etiology. Both conditions share certain clinical symptoms and abnormalities of the immune system. Patients share an increased risk for development of B cell lymphoma (1) although more pronounced in pSjD, activation of the complement system (2), epigenetically patterns of DNA methylation (3) and a prominent IFN signature well described for pSjD (4) and SLE (5). Yet their molecular and cellular drivers and details of their pathogenetic networks remain unclear.

With current developments in B cell depletion and immune modulatory therapies in autoimmunity a better understanding of differences in the immunopathogenesis and immunological alterations are crucial to improve our mechanistic understanding and subsequent successful immune targeting. Although both diseases seem to be B cell driven, clinical trials of rituximab in pSjD (6–8) and SLE (9, 10) did not consistently meet their primary endpoints. Nevertheless, B cell-directed therapies remain highly relevant, particularly in SLE, where BAFF inhibition has demonstrated clinical efficacy and newer approaches, including type II anti-CD20 antibodies, T-cell engagers, and CAR-T cell therapies, have shown promising results., These observations suggest that a more detailed understanding of the distinct underlying immune abnormalities in each disease may facilitate more effective therapeutic strategies. T cells are important players in the pathogenesis of both SLE and pSjD, primarily by driving and sustaining the autoimmune response. In both conditions, dysfunctional T cells, particularly T helper cells, are implicated in breaking immune tolerance and promoting the overproduction of autoantibodies by B cells, a process critically mediated by follicular helper T (Tfh) cells that are often expanded in both diseases (11). Innate immune cells play a crucial, initiating, and perpetuating role in both SLE and pSjD, fundamentally by driving the Type I interferon (IFN) signature, a hallmark of both diseases (5, 12). Innate cells largely comprising myeloid lineage cells are key contributors, being considered the primary producers of large amounts of IFN in both SLE and pSjD, which is typically triggered by nucleic acid-containing immune complexes or autoantigens via Toll-like receptors (TLRs), specifically TLR7 and TLR9 (13, 14). In the current study, we performed high dimensional cartography of peripheral B and T lymphocytes as well as innate immune cells of patients with pSjD, SLE, and HDs using mass cytometry. High-dimensional mass cytometry (CyTOF) permits simultaneous quantification of >20 protein markers at the single-cell level, offering unprecedented resolution to dissect immune heterogeneity. A side-by-side comparison of both adaptive and innate compartments in SLE and pSjD has not yet been reported. By integrating high-dimensional CyTOF profiling of B, T and innate compartments with consensus clustering and checkpoint receptor analyses, we provide further insights into subtle but likely important differences between pSjD and SLE.

## Materials and methods

### Study participants

A total of 15 healthy donors (HD), 21 SLE patients, and 12 pSjD patients were enrolled in this study. The study was approved by the local ethics committee of Charité Universitätsmedizin Berlin and written consent was obtained from all patients and healthy controls (EA1/302/16). Enrolled patients with pSS and SLE had an established diagnosis, characteristic autoantibody profiles, and stable disease-related medication regimens. To minimize treatment-related confounding effects, corticosteroid doses and background immunomodulatory therapies were required to be stable for at least four weeks prior to screening (**Supplementary Table 1**).

### Sample collection

Lithium Heparin (LiHep)-anticoagulated for mass cytometry measurements as well as EDTA-anticoagulated (Greiner Bio-One) blood for TBNK and flow cytometry was drawn from all patients.

### Isolation of mononuclear cells

Peripheral blood mononuclear cells (PBMCs) were isolated from Li-Hep-anticoagulated blood as described (15). Briefly, blood was mixed with PBS (Biochrom, Berlin, Germany) and subjected to density gradient centrifugation with Ficoll Paque Plus (GE Healthcare). The collected mononuclear cells were washed twice with PBS and counted prior to further studies.

### Cryopreservation of PBMCs

Up to 10x10^6^ PBMCs were transferred to FBS/10% DMSO, frozen to -80 °C in a CoolCell Freezing Container (Biocision) and stored at -80 °C until further use.

### Phenotypic analysis by CyTOF

A total of 5×10^5^ PBMCs were thawed and processed as previously described (16). The antibodies used are listed in **Supplementary Tables 2** and **3**. The two antibody panels were tested and applied separately. A Helios mass cytometry instrument (Fluidigm) was used for sample acquisition. Data analysis was performed using the Cytobank platform.

### Pre-gating of T, B lymphocytes and innate cells

Cell types were identified and gated using FlowJo (Version 10.6.1, BD). The gating strategy to identify lymphocyte subsets is shown in the **Supplementary Figures**. Fcs files of gated cells were exported for downstream clustering analysis using the CATALYST package (17).

### Cluster analysis

Clustering and dimensional reduction was performed using R and the CATALYST package (17). Fcs files of pre-gated populations of B, T or innate immune cells were joined for the three cohorts of HD, pSjD and SLE. The combined B, T or innate immune cells were than clustered via flowSOM (18) and ConsensusClusterPlus (19) algorithm using expression of cell type specific lineage and differentiation markers as parameters. The final number of clusters was selected using a combination of consensus clustering metrics like the delta area under the cumulative distribution function (CDF) and biological interpretability, aiming to resolve known immune-cell populations while avoiding over-fragmentation. Cluster and expression visualization was realized using uniform manifold approximation and projection (UMAP) for dimensional reduction (20). Clusters were manually annotated according to marker expression. Additionally, we performed multidimensional scaling (MDS) analysis to visualize overall variation of cohorts or variation between clusters.

### Determination of absolute lymphocyte counts by TBNK flow cytometry

Absolute lymphocyte subset counts were determined by flow cytometry using the BD Multitest 6-Color TBNK reagent in combination with BD Trucount tubes (BD Biosciences), according to the manufacturer’s instructions. Briefly, whole blood was stained with antibodies against CD3, CD4, CD8, CD16/CD56, CD19, and CD45 in Trucount tubes containing a known number of fluorescent counting beads. Following erythrocyte lysis, samples were acquired using a BD Canto flow cytometer. Absolute counts of CD3^+^ T cells, CD19^+^ B cells, and CD16^+^CD56^+^ NK cells were calculated based on the ratio of cellular to counting bead events and expressed as cells per microliter (cells/µl) of whole blood.

Estimated absolute counts were calculated for B cell and T cell subsets from the CyTOF clustering using donor-matched CD19^+^ B cell and CD3^+^ T cell counts obtained by TBNK flow cytometry. Equivalent calculations were not performed for innate-cell subsets because the TBNK panel provides only total CD16^+^CD56^+^ NK-cell counts and does not include absolute counts for the broader innate cell populations captured by the CyTOF panel.

### Network of correlation analysis

We combined the performed quantified relative frequencies of immune cell subsets from the clustering analysis and performed pairwise Spearman rank correlations (ρ) separately for each cohort using the SciPy package (v1.10.1). Correlations were considered for network construction if they met significance threshold of p < 0.01.

For network visualization, only correlations with an absolute effect size of |ρ| > 0.5 were used. We then constructed undirected correlation networks in igraph (v0.10.4), in which nodes represent immune cell subsets and edges represent significant correlations (blue for ρ > 0, red for ρ < 0). Node size was held constant for clarity. Differential abundance information displayed on the network was obtained from the corresponding cluster abundance analyses described above and is indicated by ↑/↓ symbols. Finally, we quantified network topology for each cohort by calculating average degree, network density, global clustering coefficient and mean betweenness centrality and compared these values across conditions.

### Expansion of B cell panel via dataset integration

To extend the B cell read-outs beyond those available in the B cell-specific panel, we also analyzed B cells from the innate panel, which includes checkpoint molecules (CPM) markers, using same-donor samples available in both panels, comprising 48 donors (14 HD, 12 pSjD, and 22 SLE). Both panels share several B cell specific and non-specific readouts, some of which are measured using the same isotope tags (209Bi_CD16, 162Dy_CD8, 163Dy_BTLA, 167Er_CD38, 168Er_Ki67, 155Gd_CD27, 156Gd_CD86, 165Ho_CD45RO, 176Lu_CD56, 144Nd_CD69, 145Nd_CD4, 146Nd_IgD, 141Pr_ICOS, 195Pt_Viability, 147Sm_CD11c, 149Sm_CD25, 159Tb_FOXP3, 169Tm_CD45RA, 89Y_CD45, 172Yb_IgM, and 174Yb_HLA-DR) and others with different tags (CD3 (Er170Di/170Er), CD14 (Gd160Di/160Gd), and CD19 (Nd142Di/142Nd)). Donor-level mean expression values were assessed and Pearson correlation coefficient were calculated for all shared markers. Markers with a correlation coefficient greater than 0.85 (BTLA, CD38, Ki67, CD27, CD86, CD45RO, IgD, ICOS, Viability, CD11c, CD25, FOXP3, CD45RA, CD45, IgM, HLA-DR, CD3, and CD19) were used for dataset integration.

Based on these identified correlations of the common read-outs we performed nearest neighbor (NN) calculations to project the CPM dataset onto the UMAP generated from the B cell-specific panel. Cluster identities in the CPM dataset were assigned according to the nearest neighboring cell in the reference dataset, thereby enabling integration of the two datasets and subsequent analysis of checkpoint molecule expression across annotated B cell populations. To evaluate the quality of the projection, the B cells cluster distributions in both the reference and projected dataset, were quantified using two metrics: the L1 distance, calculated as the sum of absolute differences between cluster proportions, and the Jensen-Shannon divergence that measures information-theoretic similarity. The metrics were calculated based on the relative frequency (proportion) of cells per cluster. In addition, cluster-specific concordance was assessed by comparing relative cluster frequencies between the reference and projected datasets on a donor-by-donor basis using Pearson and Spearman correlation analyses. Expression profiles of the clusters were correlated between the reference and projected dataset, to check for phenotypic reproducibility of the clusters.

### Checkpoint and co-stimulatory molecule expression analysis

Expression of checkpoint and co-stimulatory molecules was assessed across annotated B cell, T cell and innate immune cell clusters. Single-cell expression values were log1p-transformed and the median expression was calculated for each cluster and condition (HD. pSjD, and SLE). Heatmaps were generated using the ComplexHeatmap package (version 2.20.0). To facilitate visualization of marker-specific expression patterns, color scales were defined independently for each marker.

### Statistical analysis

Unless otherwise stated, comparisons between HD, pSjD, and SLE groups were performed using the Kruskal-Wallis test followed by Dunn’s *post hoc* test. For differential abundance analyses, Dunn’s tests were performed separately for each cluster, and p-values from pairwise group comparisons were adjusted using the Benjamini-Hochberg (BH) procedure within each cluster. For differential marker-expression analyses, Dunn’s tests were performed separately for each marker-cluster combination, and p-values were adjusted using the BH procedure within the corresponding set of pairwise comparisons. Thus, multiple-testing correction was applied within each analysis panel. Unless otherwise stated, significance annotations shown in figures correspond to BH-adjusted p-values. Statistical significance is depicted as follows: *p ≤ 0.05, **p ≤ 0.01, ***p ≤ 0.001, ****p ≤ 0.0001. Statistical analysis was performed using R project version 4.00.2.

## Results

### Study participants

Using a high-dimensional approach, we assessed the composition of the immune compartments and shifts in subset distribution including certain activation markers between SLE, pSjD and HD regarding specific subpopulations of CD19^+^ B cells, CD3^+^ T cells and various innate immune cell subsets for a better understanding of the underlying abnormalities of these autoimmune diseases. We collected blood samples from 21 SLE and 12 pSjD patients as well as 15 HD (**Supplementary Table 1**). The patient cohorts comprised predominantly female individuals with established disease and were characterized for disease activity, serological features, and current treatment exposure. Although age differed between the study groups, age was not significantly associated with immune-cell subset frequencies or participant-level median marker expression after correction for multiple testing (all FDR > 0.05), suggesting that the observed age differences were unlikely to substantially confound these readouts. Single-cell proteomics was performed using CyTOF. Before performing detailed clustering analysis, we manually gated on living single cells and the respective immune population.

### IgD^-^ CD27^-^ double negative B cell clusters are increased in patients with SLE and exhibit an activated phenotype

To identify disease dependent alterations of B cell subset distributions in SLE and pSjD, we performed a high-dimensional approach. Using consensus clustering and visualization via UMAP, we identified 13 clusters in the B cell compartment. Pre-gated B cells (**Supplementary Figure 1A**) were clustered by expression of lineage and differentiation markers CD19, CD20, CD10, CD24, CD27, CD38, CD21, CD25, HLA-DR as well as isotype expression of IgD, IgM, IgG and IgA (**Supplementary Table 2**; **Figures 1A, B**; **Supplementary Figure 1B**) to determine distribution among canonical B cell subsets and to evaluate the presence of potential novel subsets. To assess overall variation of the cohorts, we performed multidimensional scaling (MDS). We observed that while the HD samples cluster together in MDS, SLE and pSjD showed a remarkable higher variability (**Supplementary Figure 1C**) reflecting their increased heterogeneity. The delta area plot of the cumulative distribution function (CDF) of the meta-clustering suggested an appropriate range for the number of clusters between 8 to 15 (**Supplementary Figure 1D**). To maximize biological resolution and allow detection of also small but potentially relevant subpopulations, we chose 13 clusters, which we annotated (**Figure 1C**) according to their median expression profile (**Figure 1B**).

**Figure 1:**
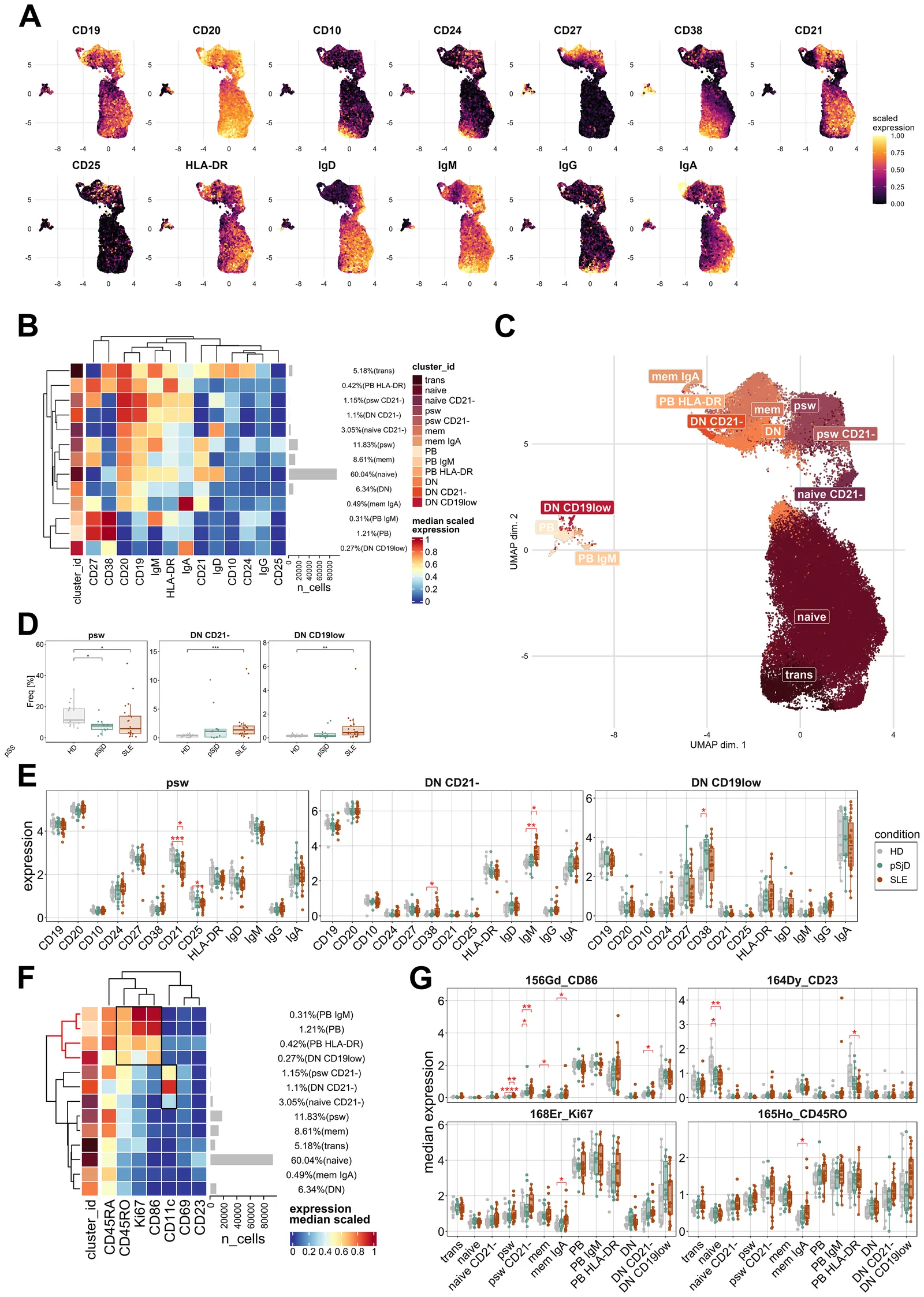
Clustering analysis reveals B cell abnormalities are more prominent in SLE than pSjD. **(A)** UMAPs of B cells showing expression of lineage and differentiation marker CD19, CD20, CD10, CD24, CD27, CD38, CD21, CD25, HLA-DR and isotypes IgD, IgM, IgG Ig A as used for clustering. **(B)** Heatmap of median scaled expression of lineage and differentiation markers per cluster. **(C)** UMAP with annotated B cell clusters. **(D)** Boxplots of relative cluster frequencies with significant differences among cohorts. **(E)** Comparison of median expression of lineage and differentiation markers of differentially abundant clusters psw, DN CD21^-^ and DN CD19^low^. **(F)** Heatmap showing expression profiles of activation markers per B cell cluster. **(G)** Comparison between HD, pSjD and SLE for differential expression of activation markers in all clusters. *Statistics:* Kruskal–Wallis with Dunn’s *post hoc* (BH correction); *p ≤ 0.05, **p ≤ 0.01, ***p ≤ 0.001, **** p ≤ 0.0001.

We identified transitional B cells (trans) as CD38, CD24 and CD10 expressing cells. Naïve B cells split up into the most abundant cluster expressing IgD^+^, CD21^+^ and CD27^-^ (naïve) and a small cluster lacking CD21 (naïve CD21^-^). We identified two pre-switched memory (psw and psw CD21^-^) clusters which differed regarding their CD21 expression. IgD^-^ CD27^+^ CD38^-^ cells are annotated as memory (mem) B cells with an additional cluster high in IgA (mem IgA) expression. Three clusters neither expressed IgD nor CD27, and therefore are double negative (DN, DN CD21^-^, DN CD19^low^) B cell subsets. PB subsets were recognized by their co-expression of CD38 and CD27 (PB, PB-HLA-DR, PB IgM) (**Figure 1C**). Notably, in the MDS analysis of the clusters, the DN CD19^low^ cluster was shifted from the other double negative clusters and aligned more closely to the PB clusters (**Supplementary Figure 1E**).

Comparing the relative abundance of the 13 B cell clusters between the cohorts we mainly found differences for SLE (**Figure 1D**; **Supplementary Figures 1F, G**). A general trend of higher relative frequencies in all PB clusters was detected and when those clusters were combined, SLE differed significantly from HD (**Supplementary Figure 1G**). While pre-switched memory B cells (psw) seem to be slightly decreased in both pSjD and SLE, the DN pool was increased in SLE. Since total DN B cells are known to be relatively expanded in patients with SLE, recent studies focus on investigating the heterogeneity of this population (21–24). The two DN clusters (DN CD21^-^ and DN CD19^low^) found to be increased in SLE in this study were both characterized by lacking Complement receptor type 2 (CR2, CD21) expression. The DN CD21^-^ cluster showed a comparable expression profile as found for the psw CD21^-^ cluster, except for low expression or absence of IgD and CD27, the other DN cluster increased in SLE showed lower CD19 expression but high IgA (**Figure 1B**) and clustered with the PBs clusters in the MDS analysis (**Supplementary Figure 1E**). Therefore, the DN CD19^low^ cluster appears to share characteristics with previously identified CXCR5^-^ CD19^low^ plasmablast precursors (24). No significant difference was found regarding the abundance of transitional, naïve, naïve CD21-, psw CD21^-^, mem, mem IgA and DN clusters between the cohorts (**Supplementary Figure 1F**).

Besides being differentially abundant in SLE, the three clusters psw, DN CD21^-^ and DN CD19^low^ also showed differences in their expression profiles. The psw cluster of SLE downregulated CD21 and CD25 surface expression, while DN CD21^-^ upregulated IgM and both DN CD21^-^ and DN CD19^low^ showed elevated CD38 expression in SLE (**Figure 1E**).

We also assessed estimated absolute abundances of CyTOF-defined B-cell subsets using donor-matched CD19^+^ B-cell counts obtained by TBNK flow cytometry (**Supplementary Figure S2A**). This analysis showed that some differences observed at the level of relative frequencies were influenced by variation in total B-cell numbers, whereas others remained evident after accounting for absolute cell counts (**Supplementary Figure S2B**). Absolute cell numbers differed between the cohorts for naïve, psw, memory, memory IgA, and total DN B cells, whereas no differences were observed for transitional, naïve CD21−, psw CD21−, PB, PB IgM, PB HLA-DR, DN CD21−, or DN CD19low B cells. Notably, the differences in DN CD21− and DN CD19low relative frequencies were therefore not accompanied by significant differences in their estimated absolute cell numbers.

It is noteworthy that the overall expression of CD21 was reduced in SLE in a variety of clusters (**Supplementary Figure S2C**). Interestingly, especially the clusters negative for CD21 (naïve CD21^-^, psw CD21^-^, DN CD21^-^ and DN CD19^low^) exhibited higher CD38 expression suggesting an activated phenotype of those clusters (**Supplementary Figure 2A**).

Besides the markers used for clustering and identifying B cell subsets, we included other relevant markers, such as activation and proliferation markers Ki-67, CD86, CD45RO, CD45RA, CD23, and CD11c (**Figures 1F, G**; **Supplementary Figure S2D**)). Clustering their expression profile revealed that the DN CD19^low^ cluster was closely associated with the PB clusters and showed an activated phenotype with elevated CD86, Ki-67 and CD45RO, while the DN CD21^-^ and psw CD21^-^ clusters exhibited increased CD11c expression (**Figure 1F**).

Comparison between cohorts showed increased CD86 expression on various clusters of patients with SLE, while CD23 expression was rather downregulated on CD23 positive clusters. The cluster mem IgA stands out by higher CD86, Ki-67 and CD45RO expression in SLE suggesting that these cells are antigen experienced and were recently activated (**Figure 1G**).

Although there were no substantial differences between the cohorts, CD11c was highest expressed on CD21^-^ clusters (**Supplementary Figure 2B**) which is in alignment with previous findings (16, 22). We found no differences regarding CD45RA expression between the cohorts.

Clustering analysis of peripheral B cells revealed disease-dependent abnormalities in both abundance and expression patterns which were more prominent in SLE suggesting a more activated B cell compartment in SLE than in pSjD.

### CD4^+^ PD-1^+^ ICOS^+^, CD8^+^ Tem and CD8^+^ Temra T cell clusters are increased in pSjD patients

Next, we analyzed the peripheral T cell compartment using a panel which included markers for detailed T subsets identification. Thus, pre-gated CD3^+^ T cells (**Supplementary Figure 3A**) were clustered by the expression of lineage markers CD4, CD8, CD27, CD24, CD45RO, CD45RA, TCRgd, CD25, HLA-DR, FOXP3, ICOS, TIGIT and PD-1 (**Supplementary Table 3**; **Figures 2A, B**; **Supplementary Figure 3B**) to determine canonical T cell subsets and sub-clusters. Regarding the variation of cohorts some patients clustered with HD while some clearly separated in the MDS analysis (**Supplementary Figure 3C**). The delta area of the CDF curve suggested an optimum of around 12 clusters (**Supplementary Figure 3D**) which we annotated (**Figure 2C**) according to their median expression profile of lineage markers (**Figures 2A, B**). Clustering of CD3^+^ T cells revealed a distinct separation between CD4 and CD8 T cells as expected (**Figure 2C**; **Supplementary Figure 3E**). We identified six CD4^+^ and four CD8^+^ as well as two CD4^-^/CD8^-^ clusters.

**Figure 2:**
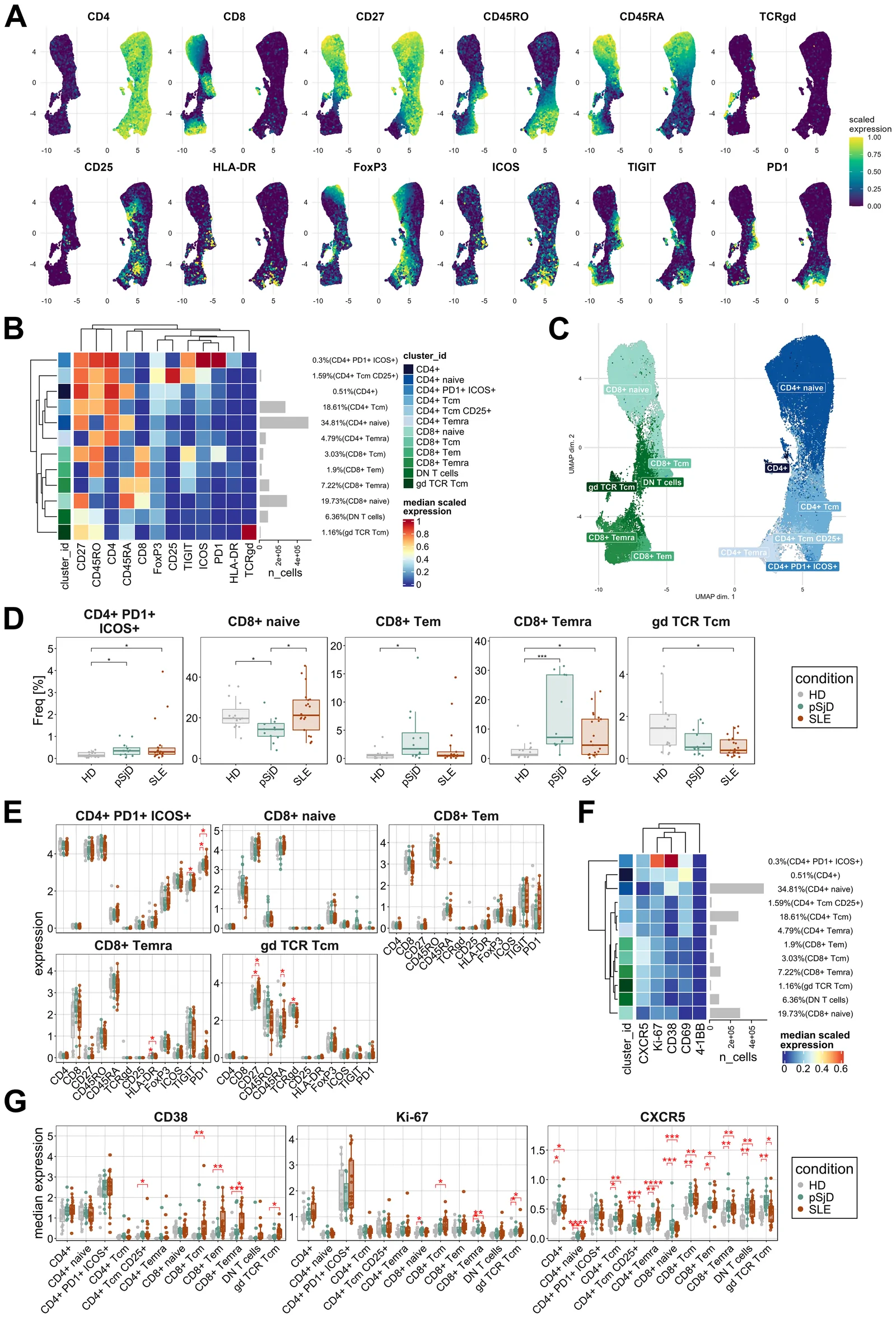
Clustering analysis reveals T cell abnormalities are more prominent in pSjD than SLE. **(A)** UMAP of T cells showing expression of lineage and differentiation marker CD4, CD8, CD27, CD24, CD45RO, CD45RA, TCRgd, CD25, HLA-DR, FOXP3, ICOS, TIGIT and PD-1 used for clustering. **(B)** Heatmap of median expression of markers per cluster. **(C)** UMAP with annotated T cell clusters. **(D)** Boxplots of relative cluster frequencies with significant differences among cohorts. **(E)** Median expression of markers of differentially abundant clusters. **(F)** Heatmap showing expression profil of activation markers CXCR5, Ki-67, CD38, CD69 and 4-1BB (CD137) per cluster. **(G)** Boxplots of median expression of significant activation markers with comparison between HD, pSjD and SLE. *Statistics:* Kruskal–Wallis with Dunn’s *post hoc* (BH correction); *p ≤ 0.05, **p ≤ 0.01, ***p ≤ 0.001, **** p ≤ 0.0001.

Although there were differences in the distribution of the 12 T cell clusters for both SLE and pSjD, we found more prominent abnormalities in pSjD (**Figure 2D**; **Supplementary Figure 3F**). The only difference in the CD4^+^ compartment was the increased frequencies of CD4^+^ PD-1^+^ ICOS^+^ cluster for patients with SLE and pSjD (**Figure 2D**). More alterations were detected in the CD8^+^ compartment. While CD8^+^ naïve T decreased, CD8^+^ Tem and CD8^+^ Temra clusters (**Figure 2D**) showed an increased proportion in pSjD. The latter was also increased in SLE but to a lesser extent than in pSjD. Additionally, we detected a decrease of CD4/CD8 double negative gd TCR Tcm in SLE (**Figure 2D**).

No significant difference was found regarding the abundance of CD4^+^, CD4+ naïve, CD4+ Tcm, CD4^+^ Tcm CD25^+^, CD4^+^ Temra, CD8^+^ Tcm and DN T cells clusters between the cohorts (**Supplementary Figure 3F**).

We also assessed estimated absolute abundances of CyTOF-defined T cell subsets using donor-matched CD3^+^ T cell counts obtained by TBNK flow cytometry (**Supplementary Figure S4A**). Although some differences were influenced by variation in total T cell numbers, the major disease-associated T cell alterations remained evident in the estimated absolute-count analysis (**Supplementary Figure S4B**).

Evaluation of disease-dependent differences in lineage marker expression found that CD4^+^ PD-1^+^ ICOS^+^ cluster representing circulating T follicular helper cells expressed more TIGIT in SLE and more PD-1 in both SLE and pSjD (25). Interestingly, although CD8^+^ naïve/Tem/Temra clusters showed differences in relative frequency they did not differ regarding expression levels or profile of lineage markers besides an increase of HLA-DR in the CD8^+^ Temra cluster. The gd TCR Tcm cluster upregulated CD27 expression in pSjD and SLE while expression of the gdT cell receptor was decreased in SLE (**Figure 2E**). In general, TIGIT, ICOS and PD-1 were upregulated on certain pSjD and SLE clusters (**Supplementary Figure 4C**).

Furthermore, we checked the expression levels of additional markers of the T cell panel such as CXCR5, Ki-67, CD38, CD69, and 4-1BB (CD137) (**Figures 2F, G**; **Supplementary Figure 4D**). Of note, the CD8+ memory T cell clusters in SLE upregulated CD38 expression. The proliferation marker Ki-67 was found upregulated in SLE for CD8^+^ Tcm and in pSjD for CD8^+^ naïve while it was upregulated in both conditions for CD8^+^ Temra and gd TCR Tcm.

Of particular interest, we found antigen-experienced CD8^+^ T cell subsets were enhanced in patients, especially in pSjD and accounted for a diminished naive compartment.

### Innate immune cell profiling reveals subset-specific activation and regulatory marker alterations in pSjD versus SLE

Gated as non-B or T cells (**Supplementary Figure 5A**), innate immune cells such as monocytes (M), dendritic cells (DC) and natural killer (NK) cells were clustered and identified based on the expression of the following markers: CD14, CD16, CD56, CD86, CD27, CD123, CD11c, CD226, HLA-DR and CD4 (**Supplementary Table 3**; **Figures 3A, B**; **Supplementary Figure S5B**). In alignment with the delta plot (**Supplementary Figure 5C**) which suggests 10–14 clusters, we identified and annotated 13 clusters (**Figures 3B, C**) of innate immune cells according to their median expression profile (**Figure 3B**). Six of the clusters were assigned to monocytes based on CD4, CD14, CD16, CD11c and HLA-DR expression. Classical monocytes (CM) and intermediate monocytes (IM) were each divided into two subsets according to their CD226 expression. Additional monocyte clusters are CD14^-^/CD16^++^ non-classical monocytes (NCM) and a cluster named CD226^hi^ M. This cluster was characterized by intermediate expression of CD14 and CD16 and a general low expression profile except high CD226. This cluster was the least abundant (**Figure 3C**) and clustered separately from the rest of the monocyte clusters in UMAP and MDS plots (**Supplementary Figure 5E**).

**Figure 3:**
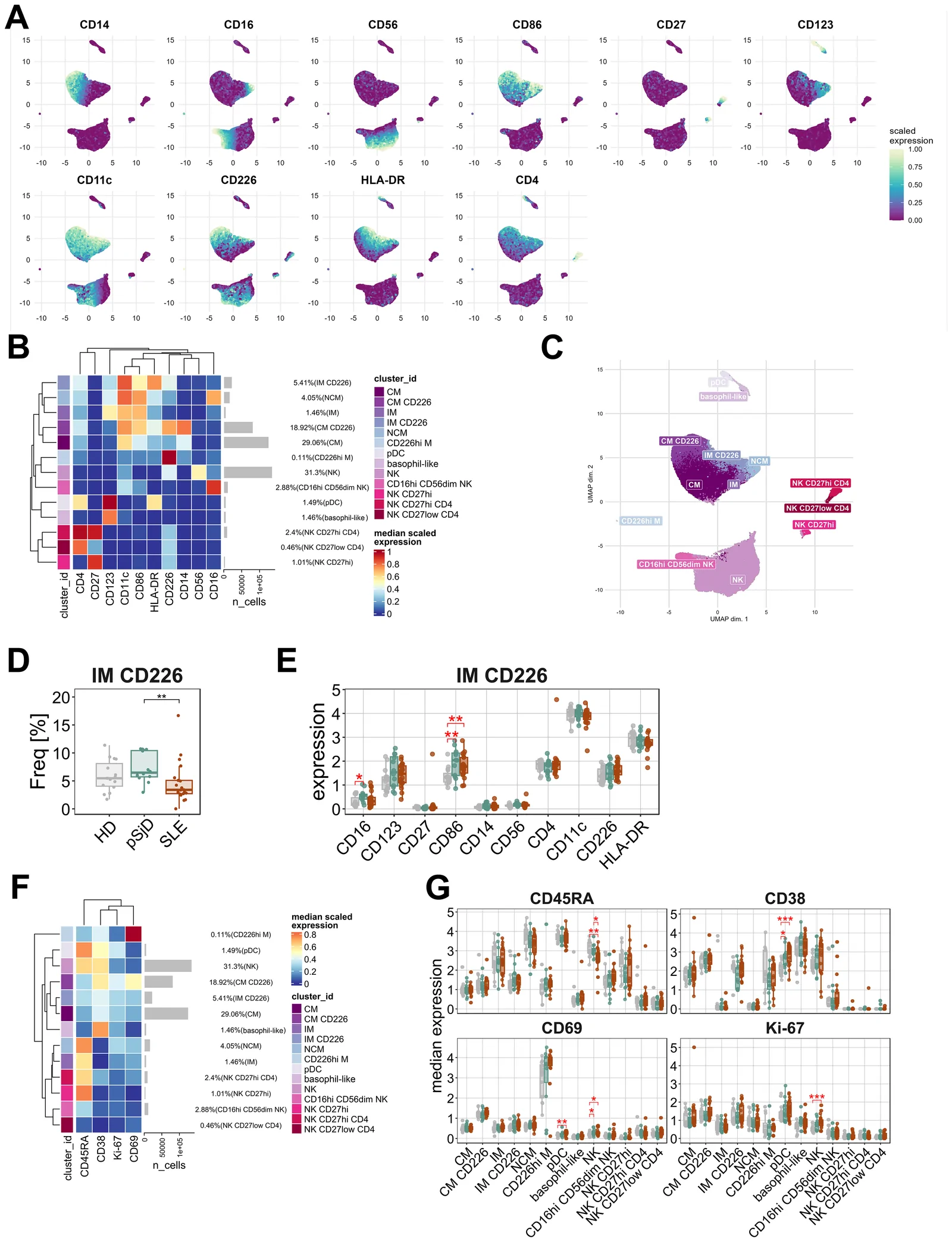
Innate immune cell profiling reveals subset-specific activation and regulatory marker alterations in pSjD and SLE. **(A)** UMAP of innate cells showing expression of lineage marker CD14, CD16, CD56, CD86, CD27, CD123, CD11c, CD226, HLA-DR and CD4 used for clustering. **(B)** Heatmap of median expression of markers per cluster. **(C)** UMAP with annotated innate cell clusters. **(D)** Boxplots of relative cluster frequencies with significant difference among cohorts. **(E)** Median expression of markers of differentially abundant clusters. **(F)** Heatmap showing expression profil of activation markers CD45RA, CD38, Ki-67 and CD69 per cluster. **(G)** Boxplots of median expression of activation marker with comparison between HD, pSjD and SLE. *Statistics:* Kruskal–Wallis with Dunn’s *post hoc* (BH correction); *p ≤ 0.05, **p ≤ 0.01, ***p ≤ 0.001, **** p ≤ 0.0001.

The two CD123bright clusters were identified as plasmacytoid (pDC) and basophil-like. The rest of the clusters are of natural killer phenotype (**Figures 3B, C**).

Clustering the samples based on the innate cell data indicated that samples from HD, pSjD, and SLE do not form distinct groups, but instead overlap significantly (**Supplementary Figure 5D**). In alignment with that, the cluster IM CD226 was the only one that differed significantly in abundance between cohorts, with a reduced relative frequency in SLE compared to pSjD (**Figure 3D**). The frequencies of all the other clusters did not differ significantly between the cohorts (**Supplementary Figure 5F**).

Regarding expression of various molecules, IM CD226 cluster upregulate CD86 expression in both pSjD and SLE. Additionally, a slight increase of CD16 expression by patients with pSjD compared to HDs was detected (**Figure 3E**).

The expression profile for all clusters did not reveal substantial cohort dependent differences. Nevertheless, it is noteworthy, that especially the IM and NCM clusters of patients with SLE presented with higher CD86 expression which allows the conclusion they are of an activated phenotype. HLA-DR expression was increased in the IM cluster of patients with pSjD (**Supplementary Figure S6A**).

Furthermore, we checked the expression levels of additional markers which included CD45RA, CD45RO, CD25, CD38, CD69, TCRgd, 4-1BB (CD137) and Ki-67 (**Figures 3F, G**; **Supplementary Figure 6B, C**). We found that NK cells and DCs exhibited signs of activation. CD45RA expression was decreased in NK cells in both pSjD and SLE, pointing to a shift toward an activated phenotype. Further evidence for activation of NK cells was found by the elevated CD69 expression in both pSjD and SLE, while Ki-67 expression was solely increased in SLE patients (**Figure 3G**). The pDCs cluster also displayed features of activation with increased CD38 expression for both pSjD and SLE. Additionally, CD69 expression was increased in pDCs from SLE, albeit at very low levels (**Figure 3G**).

CD45RO, CD25, 4-1BB (CD137) and TCRgd were mostly expressed at very low levels and, interestingly, no difference was found for those markers between cohorts (**Supplementary Figure 6D**).

### Correlation analysis reveals distinct patterns of immune cell co-variation in pSjD and SLE

To further explore the co-regulation patterns between the frequencies of the different immune cell subsets and immune compartments across and within each condition, we performed a correlation analysis (**Supplementary Figure 7**) that combined frequencies of B cell, T cell, and innate cell subsets. The resulting correlation networks (**Figures 4A, B**) revealed distinct correlation patterns of immune-cell co-variation within the different conditions, using a threshold of Spearman’s ρ > 0.5 and p < 0.01,

**Figure 4:**
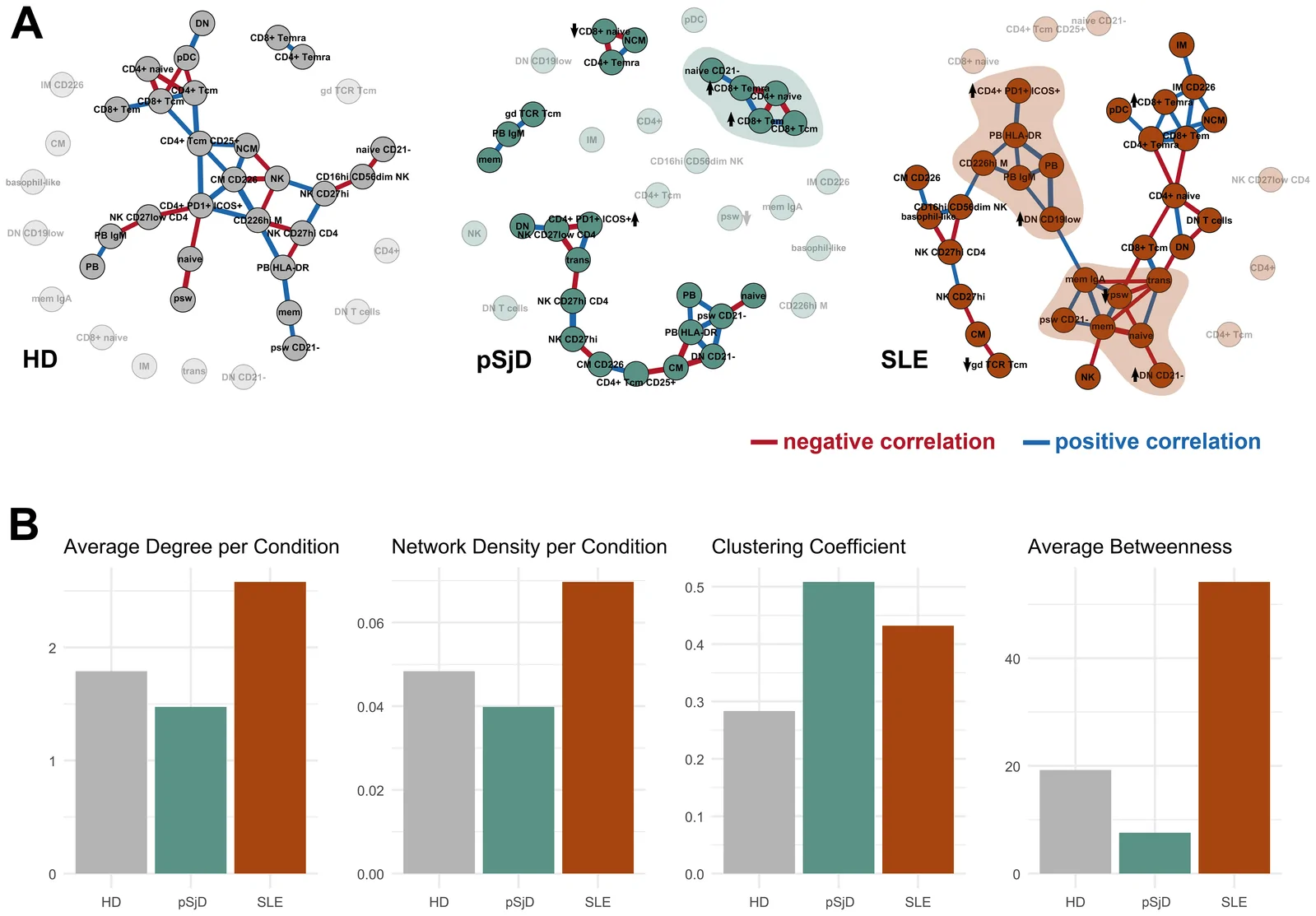
Correlation networks of relative immune cell subset frequencies. **(A)** Network graphs depict significant Spearman correlations (p < 0.01) between relative immune cell subset frequencies for the HD cohort, the pSjD cohort or the SLE cohort. Nodes represent immune cell subsets, edges represent negative (red) or positive (blue) correlations. Arrows indicate immune cell subsets with significantly increased (↑) or decreased (↓) frequencies within each condition. Shaded areas highlight areas of interest in pSjD and SLE. **(B)** Quantitative network metrics per condition. The four bar plots depict average degree, network density, clustering coefficient, and average betweenness centrality for each condition.

A general observation was that we found a higher connectivity in SLE than pSjD, whereas pSjD displayed more separated correlation patterns with only specific immune subsets correlating together. This might reflect a more tightly linked regulation of immune cell alterations across all immune compartments in SLE. Healthy donors exhibited intermediate connectivity, with average degree and network density values between those observed in pSjD and SLE. In pSjD, a notable pattern of cytotoxic T cells including CD8^+^ Tcm, and the relatively expanded clusters CD8^+^ Tem and CD8^+^ Temra all correlating inversely with the CD4+ naïve T cell cluster. Interestingly, the CD8^+^ Temra cluster correlated with naive B cells lacking CD21 expression (naive CD21^-^), suggesting a co-regulation of cluster frequencies between antigen-experienced T cells and atypical B cells in pSjD (**Figure 4A**). Although the overall relative frequency was not increased in pSjD, variations of plasmablast clusters (PB, PB HLA-DR) frequencies within the pSjD cohort correlated with frequencies of CD21 negative B cell clusters (psw CD21, DN CD21^-^) (**Figure 4A**).

The SLE network exhibited a widespread interconnection reflected by the identified correlations as well as some distinct patterns with a dense structure (**Figure 4B**) of various subsets correlating. Of interest, subsets increased in SLE, such as PB clusters (PB, PB HLA-DR), the DN CD19^low^ cluster, and activated CD4^+^ T cells (CD4^+^ PD-1^+^ ICOS^+^) showed strong correlations with each other, forming a group of subsets whose frequencies tend to shift together across individuals within this particular cohort. Additionally, this pattern showed a linkage of DN CD19^low^ and mem IgA connected to another dense pattern of co-varying memory and naïve B cell clusters. Importantly, innate cell subsets (pDCs, IM and NCM) also showed correlations with effector T cell populations (CD8^+^ Temra, CD8^+^ Tem, CD4^+^ Temra), suggesting that relative frequency changes in SLE correlate across multiple immune compartments with various correlations observed for fluctuating memory B cell and plasmablast clusters.

### CPM profiling shows altered expression pattern of costimulatory receptors in SLE

Next, we investigated disease-specific immune checkpoint signatures across cell types and conditions. Therefore, we integrated the CPM and B cell-specific panel. Subsequently, we calculated correlations for their shared readouts (**Supplementary Figures 8A, B**), then used these relationships to perform nearest‐neighbor (NN) mapping (**Supplementary Figure 9A**). This approach permitted projection of CPM measurements onto the B cell reference clusters, enabling CPM analysis of the described B cell clusters (**Supplementary Figure 9B**). To evaluate how well the projected data represents the reference distribution, we compared cluster frequencies between the CPM‐projected and B cell reference datasets. The L_1_ distance between their cluster‐proportion vectors was 0.165, while the Jensen–Shannon divergence was 0.0098, indicating a high degree of similarity between the reference and projected distributions (**Supplementary Figure 9B**). In addition, cluster-specific validation showed strong concordance between the reference and projected datasets, Comparison of relative cluster frequencies demonstrate high agreement across most B cell populations, including plasmablast subsets (PB: r = 0.92; PB IgM: r = 0.90; PB HLA-DR: r = 0.78) and DN CD19^low^ B cells (r = 0.76), whereas lower concordance was observed for the rare mem IgA population (**Supplementary Figure 9C**). Furthermore, comparison of cluster-specific marker expression profiles demonstrated high concordance between reference and projected clusters (Pearson r = 0.85–1.00), including plasmablast (r = 0.99) and DN CD19low (r = 0.93) subsets, supporting preservation of cluster identity following nearest-neighbor projection (**Supplementary Figure 9D**). Consistent with these findings, side-by-side UMAPs depict highly similar marker expression patterns between reference cells from the B cell panel and projected query cells from the innate panel (**Supplementary Figure 9E**).

To gain further insight into the checkpoint molecule expression across immune cell subsets, we compared the mean expression levels of inhibitory and co-stimulatory receptors including BTLA, TIGIT, CTLA-4, LAG-3, VISTA, PSGL-1 and PD-1 and costimulatory receptors like ICOS, CD226, CD86, CD155 in B, T, and innate cell subsets from HD, pSjD, and SLE (**Figure 5**).

**Figure 5:**
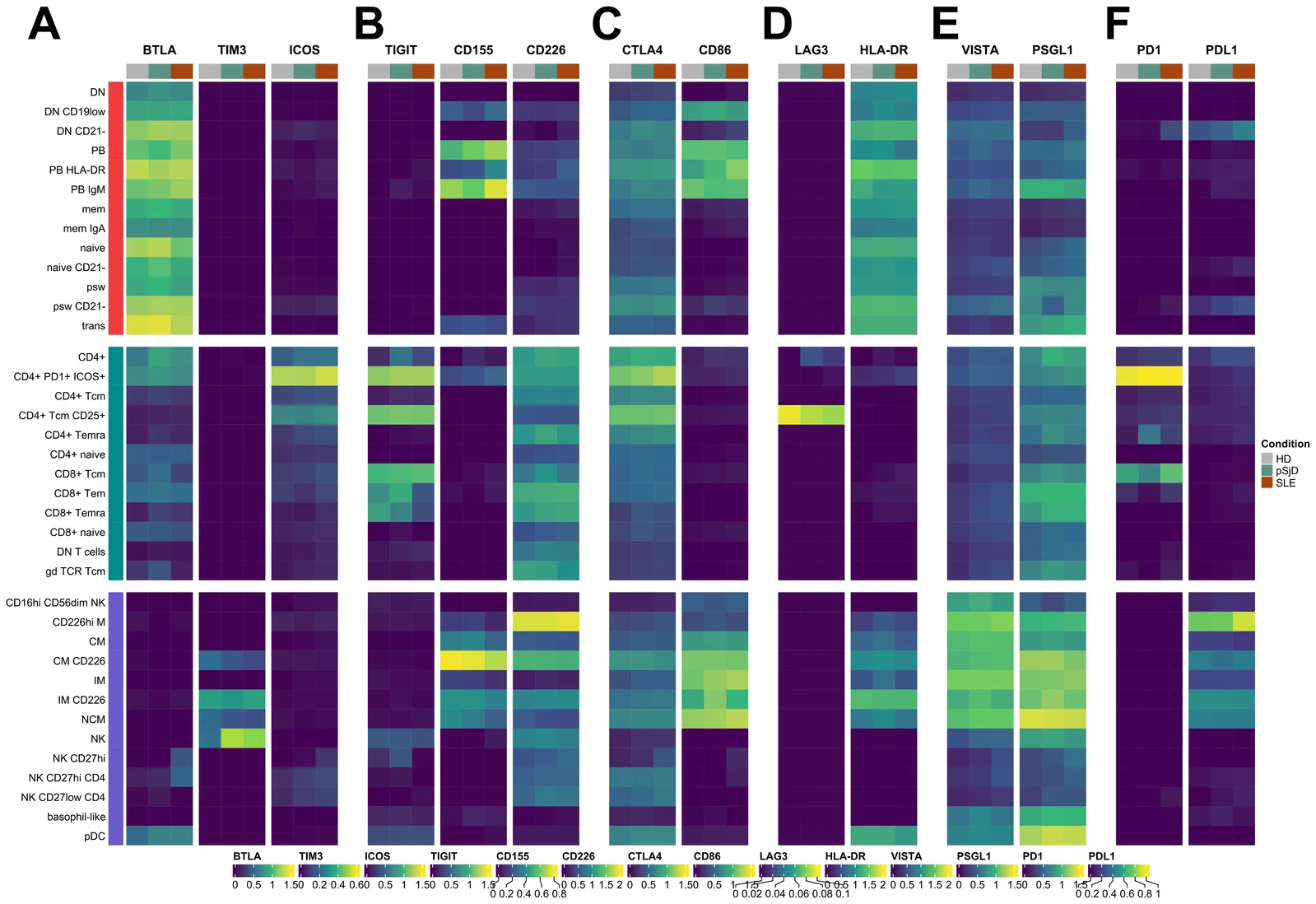
Checkpoint molecule expression defines distinct immune compartment signatures. Heatmap depicting median expression of inhibitory and co-stimulatory receptors across B cell (red), T cell (green), and innate immune (purple) clusters. Rows correspond to immune subsets, and columns represent checkpoint molecules across HDs, pSjD and SLE. Expression values are log1p-transformed and summarized as cluster-level medians. Color scales are defined separately for each marker to facilitate visualization of marker-specific expression patterns. Therefore, the color scale indicates relative expression intensity, from low (blue) to higher expression (yellow). Top annotation bars indicate the sample condition. Depicted are: **(A)** BTLA, TIM3, ICOS **(B)** TIGIT, CD155, CD226 **(C)** CTLA4, CD86, **(D)** LAG3, HLA-DR, **(E)** VISTA, PSGL1 and **(F)** PD-1, PD-L1.

BTLA was predominantly expressed across B cell subsets, in a differentiation‐dependent pattern: highest in naïve B cells, downregulated in classical memory, and re‐upregulated in plasmablasts (**Figure 5A**). Disease-associated alterations were minimal, aside from modest BTLA reduction in SLE gdTCR Tcm cells. ICOS was selectively elevated in SLE on CD4^+^ naïve, Tcm, and DN clusters (**Figure 5A**; **Supplementary Figure S10A**).

Next, we checked TIGIT, an inhibitory receptor and its corresponding ligand CD155 (26). TIGIT was not found on B cells and only slightly expressed by some innate clusters and showed disease-associated changes primarily in CD4^+^ memory T cells (**Figure 5B**), where both pSjD and SLE increased TIGIT on CD4^+^ Tcm cells (**Supplementary Figure S10B**). pSjD exhibited broader TIGIT enhancement across CD4^+^ subsets, while SLE selectively increased TIGIT on CD4^+^PD-1^+^ICOS^+^ and CD4^+^ Temra cells. CD226, the costimulatory competitor of TIGIT (27), showed SLE-specific upregulation on transitional and memory B cells and on CD226^hi^ monocytes, whereas pSjD showed more limited changes.

CTLA-4 levels on B cells did not differ between conditions but were increased in SLE CD4^+^ and DN T cells and in pSjD CD8^+^ naïve T cells. Its ligand CD86 was strongly upregulated in SLE across multiple antigen-experienced B-cell states (psw, psw CD21^−^, mem, mem IgA, DN and DN CD21^-^). In contrast, circulating T cells did not show any CD86 modulation between the cohorts (**Figure 5C**; **Supplementary Figure S10C**).

LAG-3 remained nearly absent on circulating lymphocytes, and no consistent disease modulation was detected (**Figure 5D**; **Supplementary Figure S10D**). HLA-DR as functional partner of LAG-3 was slightly upregulated in SLE in the CD8^+^ Tcm and Temra cluster.

VISTA was upregulated across all T-cell clusters in both diseases and selectively increased on CD21^−^ and plasmablast B cells (SLE > pSjD). Innate cells—including monocytes, basophil-like, and NK subsets—displayed robust VISTA increases in pSjD and SLE. PSGL-1 was preferentially elevated in pSjD, particularly in naïve and DN T-cell subsets, while showing a more restricted pattern in SLE (**Figure 5E**; **Supplementary Figure S10E**).

The PD-1/PD-L1 axis was most prominently activated in CD4^+^ memory and CD4^+^ PD-1^+^ ICOS^+^ T cells in both diseases. On B cells, PD-1 and PD-L1 increased specifically on CD21^−^ psw and DN B cells, with stronger PD-L1 upregulation in few SLE patients. Monocytes expressed PD-L1 but with no difference in disease (**Figure 5F**; **Supplementary Figure S10F**).

While most checkpoint receptors showed low baseline expression in healthy B cells and T cells, disease‐specific modulations were most pronounced in SLE, particularly for costimulatory molecules. SLE was characterized by upregulation of costimulatory molecules (ICOS on T cells, CD86 on B cells, CD226 on B cells/monocytes). Autoimmune‐associated upregulation of inhibitory receptors (e.g., TIGIT, VISTA) and ligands (e.g., PD-L1) highlights potential targets for immunomodulation in both pSjD and SLE.

## Discussion

Our high-dimensional analysis using mass cytometry enabled detection of subtle phenotypic shifts across diverse immune cell types in patient vs healthy cohorts. The analysis of B cells, T cells, and innate immune cells in pSjD, SLE patients and HD revealed that both diseases exhibit enhanced activation across adaptive and innate compartments. Additionally, it demonstrated both shared and disease-specific immune alterations between the two autoimmune diseases.

Consistent with previous reports, we observed increased relative frequencies of double-negative (DN) B cells, particularly the DN CD21^−^ (16, 22, 28) and DN CD19^low^ subsets (24), in patients with SLE, suggesting expansion of atypical or exhausted B cells associated with chronic activation and autoantibody production. The DN CD19^low^ subset also exhibited high IgA and CD38 expression, clustering near plasmablasts and resembling CD19^low^ CXCR5^−^ precursors of antibody-secreting cells (24). The expansion of IgA^+^ cells is a notable finding and is consistent with earlier reports (29) demonstrating increased IgA^+^ plasmablasts carrying signatures of mucosal induction. Elevated Ki-67 and CD86 expression in DN and memory B cell subsets further support their activated phenotype in SLE.

In contrast, pSjD only showed a decrease of psw memory B cells in the B cell compartment. T cell alterations were more pronounced in pSjD, particularly within the CD8^+^ compartment, where CD8^+^ Tem and CD8^+^ Temra subsets exhibited increased relative frequencies. This suggests a shift towards antigen-experienced cytotoxic populations possibly driven by persistent viral or autoantigen stimulation and aligning with previous reports linking CD8^+^ memory T cells to persistent immune activation in pSjD (30–32).

The CD4^+^ PD-1^+^ ICOS^+^ T cell cluster exhibited increased relative frequencies in both SLE and pSjD. This subset showed elevated expression of TIGIT and ICOS in SLE, two markers linked to chronic T cell activation and impaired regulation (33). In addition, the population expressed CXCR5, although at moderate levels, supporting its classification as a T follicular helper-like population rather than a definitive cTfh subset. Consistent with previous studies, Tfh-like cells have been implicated in supporting autoreactive B-cell maturation and immunoglobulin class switching in autoimmune diseases (34).

Though relative frequencies of most innate immune subsets did not significantly differ between groups, phenotypic profiling revealed disease-specific activation. IM CD226 monocytes were significantly reduced in SLE, while both IM and NCM subsets displayed increased CD86 expression. This observation is in line with the reported systemic monocyte activation in SLE (35). In both diseases, pDCs exhibited increased CD38 expression, with additional CD69 upregulation in SLE. NK cells showed signs of both activation and exhaustion, with decreased CD45RA in pSjD and SLE and altered checkpoint receptor expression, including increased CD155 in SLE and TIM-3 in pSjD. A trend of increased VISTA expression across multiple innate and adaptive subsets suggests widespread immune regulatory changes in both diseases. Upregulation of VISTA could reflect a counter regulating effect, since loss of VISTA can lead to autoimmunity (36). Recent studies of multiple sclerosis lesions and experimental autoimmune encephalomyelitis have shown that VISTA expression by microglia decreases with inflammation, thus supporting its regulatory role (37, 38).

We note that changes in CD14 expression associated with inflammatory states may contribute to phenotypic heterogeneity within the monocyte compartment and could influence the precise boundaries between some innate cell populations. While the overall disease-associated patterns were robust across clustering resolutions, future studies using complementary approaches will be valuable to further refine the characterization of these populations.

Because lymphopenia is common in both SLE and pSjD, interpretation of immune-cell subset frequencies may be influenced by changes in total cell numbers. We therefore complemented our frequency-based analyses with estimated absolute counts derived from donor-matched TBNK measurements. While some differences were attenuated after accounting for total lymphocyte abundance, several key disease-associated alterations remained evident, indicating that both changes in immune-cell composition and overall lymphocyte numbers contribute to the observed immune phenotypes.

Finally, both SLE and pSjD are clinically heterogeneous diseases. Although patients were enrolled under stable treatment conditions and characterized with respect to disease activity and serological characteristics, the available cohort size did not permit stratification according to specific clinical manifestations, disease duration, or molecular subtypes. Future studies in larger and more deeply phenotyped cohorts will be important to determine how the identified immune signatures relate to distinct clinical and immunological disease subsets. Network analysis revealed distinct immune connectivity patterns.

The correlation network analysis should be interpreted as exploratory and hypothesis-generating, as correlation-based networks do not establish causal relationships and may be influenced by cohort size and inter-individual variability. Patients within the SLE cohort displayed a hyperconnected immune network with high density, average degree, and betweenness centrality, supporting the hypothesis of a widespread immune activation. The SLE network linked DN B cells, plasmablasts, and activated T cells (CD4^+^ PD-1^+^ ICOS^+^), reinforcing the interplay between B and T cells in sustaining disease activity. In contrast, the pSjD cohort exhibited a more compartmentalized network. The observed inverse correlation between CD8^+^ memory T cells and CD4^+^ naive T cells in pSjD suggests a shift toward antigen-experienced populations, thus potentially reflecting chronic activation. Future studies in independent cohorts and functional studies will delineate whether these correlations reflect direct biological interactions.

Investigating the CPM expression levels revealed an upregulation of co-stimulatory pathways in SLE. ICOS was increased on CD4^+^ T cell subsets (naïve, Tcm, Temra, DN), CD86 was induced on pre-switched, switched memory, and DN B cells, and CD226 was elevated on transitional, naïve CD21^−^, memory B cells, and CD226^hi^ monocytes in SLE.

In pSjD, co-stimulatory receptors were rather unchanged in the periphery, while inhibitory checkpoints were upregulated. TIGIT on CD4^+^ Tcm/clusters, VISTA on DN B cells and all T cells, and PSGL-1 on naïve, DN, CD21^−^ B cells and various T cell subsets may indicate an intrinsic counter regulation. Overall, our integrated single‐cell proteomic profiling using mass cytometry delineates immune signatures in pSjD and SLE. SLE is characterized by B cell activation, plasmablast expansion, systemic immune activation and upregulation of costimulatory molecules (ICOS, CD86, CD226), whereas pSjD is characterized by cytotoxic T‐cell enrichment and upregulation of inhibitory checkpoints (TIGIT, VISTA, PSGL-1). These insights refine our understanding of the abnormalities in the immune compartments in pSjD and SLE and highlight pathways that may warrant further investigation. The immune signatures identified in SLE, particularly the expansion of DN B cells and activation of the Tfh–B-cell costimulatory axis, may help guide future studies aimed at identifying disease-relevant cellular and molecular targets. The increased expression of inhibitory checkpoints in pSjD highlights these pathways as candidates for future functional investigation. Future studies should validate these findings in larger cohorts and explore their functional implications.

## Limitations and future directions

This study is limited by its modest cohort size, particularly in the pSjD group, and its cross-sectional design, which precludes assessment of temporal dynamics and causal relationships. In addition, the checkpoint molecule analysis relied on integration of two CyTOF panels, although multiple validation approaches supported the robustness of the mapping procedure. Finally, the biological significance of the identified immune signatures remains to be established through functional studies. Future work in larger, longitudinal cohorts will be important to validate and extend these findings.

## Data Availability

The raw data supporting the conclusions of this article will be made available by the authors, without undue reservation.
