# Supplementary material for: Single-cell mass cytometry cartography highlights systemic lupus erythematosus and Sjögren’s disease immune differences
Source: Front Immunol. 2026 Oct 1;17:1834700. doi: 10.3389/fimmu.2026.1834700 (PMC13669203; doi:10.3389/fimmu.2026.1834700)
Supplement: Supplementary file 1 [file Table1.pdf]

Supplementary Table S1: Patient demographics and clinical characteristics

	**pSjD**	**SLE**	**HD**
	n=12	n=20	n=14
Demographic			
age (y, mean±SD)	53.6 ± 14.5	41.7 ± 12.4	33.3 ± 10.7
female % (n)	92% (11)	90% (18)	93% (13)
Disease activity			
current disease activity (mean±SD)	ESSDAI 2.33 ± 2.31	SLEDAI 4.05 ± 3.55	n.a.
Autoantibody profiles			
ANA positivity % (n), range	91.7% (11/12) 1:160 - 1:10240	100% (19/19) 1:640 - 1:5120	n.a.
Anti-dsDNA % (n)	-	Ever pos: 84.2% (16/19)	n.a.
Anti-Sm % (n)	pos: 0.0% (0/11)	pos: 10.5% (2/19)	n.a.
Anti-Ro (SS-A) % (n)	pos: 90.9% (10/11)	pos: 73.7% (14/19)	n.a.
Anti-La (SS-B) % (n)	pos: 36.4% (4/11)	pos: 36.8% (7/19)	n.a.
Current medication			
w/o systemic therapy % (n)	16.7% (2)	10% (2)	n.a.
Prednisolone % (n)	33.3% (4)	55.0% (11)	n.a.
Hydroxychloroquine % (n)	66.7% (8)	55.0% (11)	n.a.
Mycophenolate mofetil % (n)	-	20.0% (4)	n.a.
Azathioprine % (n)	-	15.0% (3)	n.a.
Methotrexate % (n)	-	15.0% (3)	n.a.
Belimumab % (n)	-	10.0% (2)	n.a.
Immune cell frequencies and counts			
**CD19+ B cells**			
frequency (%, mean±SD)	11% ± 5.6	12.4% ± 8,1	14.4% ± 2.8
absolute count (cells/µl, mean±SD)	195.6 ± 133.7	153.8 ± 163,8	313.3 ± 92.4
**CD4+ T cells**			
frequency (%, mean±SD)	38.5% ± 13.4	30.7% ± 10.1	38.6% ± 15.8
absolute count (cells/µl, mean±SD)	643.8 ± 340.7	348.1 ± 179.0	866.2 ± 491.9
**CD8+ T cells**			
frequency (%, mean±SD)	34.9% ± 13.5	44.7% ± 13.6	33.4% ± 15.0
absolute count (cells/µl, mean±SD)	556 ± 230.9	544.9 ± 330.8	744.3 ± 404.7
**CD16+CD56+ NK cells**			
frequency (%, mean±SD)	12.2% ± 5.1	9.3% ± 5.0	10.0% ± 3.6
absolute count (cells/µl, mean±SD)	182.2 ± 55.8	102.5 ± 52.5	232.6 ± 142.9
**Siglec-1** on CD14+ monocytes (MFI, mean±SD)	1176.6 ± 1329.3	1854.4 ± 1155.5	367.9 ± 112.8
**Plasmablasts** (% of B cells, mean±SD)	1.7% ± 1.1	5.0% ± 7.8	1.0% ± 0.6
