# Supplementary material for: Single-cell mass cytometry cartography highlights systemic lupus erythematosus and Sjögren’s disease immune differences
Source: Front Immunol. 2026 Oct 1;17:1834700. doi: 10.3389/fimmu.2026.1834700 (PMC13669203; doi:10.3389/fimmu.2026.1834700)
Supplement: Supplementary file 2 [file Table2.pdf]

Supplementary Table S2: B cell panel

label	Target	Manufacturer	pre-gating	B cell lineage marker	B cell activation marker
Bi209Di	**CD16**	**Fluidigm**	x		
Dy162Di	**CD8**	**Fluidigm**	x		
Eu151Di	**CD14**	**Fluidigm**	x		
Ir191Di	**DNA1**	**Fluidigm**	x		
Ir193Di	**DNA2**	**Fluidigm**	x		
Nd145Di	**CD4**	**Fluidigm**	x		
Pt195Di	**Viability**	**Fluidigm**	x		
Tb159Di	**FoxP3**	**Fluidigm**	x		
Y89Di	**CD45**	**Fluidigm**	x		
Yb170Di	**CD3**	**Fluidigm**	x		
Lu176Di	**CD56**	**Fluidigm**	x		
Ce142Di	**CD19**	**Fluidigm**	x	x	
Dy160Di	**IgG**	**Fluidigm**		x	
Dy161Di	**IgA**	**Fluidigm**		x	
Er166Di	**CD24**	**Fluidigm**		x	
Er167Di	**CD38**	**Fluidigm**		x	
Gd155Di	**CD27**	**Fluidigm**		x	
Gd158Di	**CD10**	**Fluidigm**		x	
Nd146Di	**IgD**	**Fluidigm**		x	
Sm149Di	**CD25**	**Fluidigm**		x	
Sm152Di	**CD21**	**Fluidigm**		x	
Yb171Di	**CD20**	**Fluidigm**		x	
Yb172Di	**IgM**	**Fluidigm**		x	
Yb174Di	**HLA-DR**	**Fluidigm**		x	
Dy163Di	**BTLA**	**Fluidigm**			x
Dy164Di	**CD23**	**Fluidigm**			x
Er168Di	**Ki67**	**Fluidigm**			x
Gd156Di	**CD86**	**Fluidigm**			x
Ho165Di	**CD45RO**	**Fluidigm**			x
Sm147Di	**CD11c**	**Fluidigm**			x
Tm169Di	**CD45RA**	**Fluidigm**			x
Nd144Di	**CD69**	**Fluidigm**			
Pr141Di	**ICOS**	**Biolegend**			
