# Supplementary material for: Single-cell mass cytometry cartography highlights systemic lupus erythematosus and Sjögren’s disease immune differences
Source: Front Immunol. 2026 Oct 1;17:1834700. doi: 10.3389/fimmu.2026.1834700 (PMC13669203; doi:10.3389/fimmu.2026.1834700)
Supplement: Supplementary file 3 [file Table3.pdf]

Supplementary Table S3: T/ innate Cytof panel

label	Target	Manufacturer	pre-gating	T cell: lineage marker	T cell: activation marker	innate immune cells: lineage marker	innate immune cells: activation marker
Er170Di	CD3	Fluidigm	x				
Nd142Di	CD19	Fluidigm	x				
Nd146Di	IgD	Fluidigm	x				
Pt195Di	viability	Fluidigm	x				
Y89Di	CD45	Fluidigm	x				
Yb172Di	IgM	Fluidigm	x				
Gd155Di	CD27	Fluidigm		x		x	
Nd145Di	CD4	Fluidigm		x		x	
Yb174Di	HLA-DR	Fluidigm		x		x	
Eu153Di	Tigit	Fluidigm		x			x
Ho165Di	CD45RO	Fluidigm		x			x
Lu175Di	PD1	Fluidigm		x			x
Sm149Di	CD25	Fluidigm		x			x
Sm152Di	TCRgd	Fluidigm		x			x
Tm169Di	CD45RA	Fluidigm		x			x
Dy162Di	CD8	Fluidigm		x			
Pr141Di	ICOS	Biolegend		x			
Tb159Di	FoxP3	Fluidigm		x			
Yb171Di	CD226	Fluidigm			x	x	
Gd156Di	CD86	Fluidigm				x	
Bi209Di	CD16	Fluidigm				x	
Eu151Di	CD123	Fluidigm				x	
Gd160Di	CD14	Fluidigm				x	
Lu176Di	CD56	Fluidigm				x	
Sm147Di	CD11c	Fluidigm				x	
Dy163Di	BTLA	Fluidigm			x		x
Er166Di	CD155	Biolegend			x		x
Er167Di	CD38	Fluidigm			x		x
Er168Di	Ki-67	Fluidigm			x		x
Gd154Di	TIM3	Fluidigm			x		x
Gd158Di	4-1BB	Fluidigm			x		x
Nd143Di	VISTA	Roche			x		x
Nd144Di	CD69	Fluidigm			x		x
Nd148Di	PDL1	Fluidigm			x		x
Dy161Di	CTLA4	Fluidigm			x		
Dy164Di	CXCR5	Fluidigm			x		
Nd150Di	LAG3	Fluidigm			x		
Yb173Di	PDGL1	Biolegend			x		
